# The Uptake of Nickel-Titanium Rotary Files in Saudi Arabia

**DOI:** 10.1155/2012/484291

**Published:** 2012-04-10

**Authors:** Emad AlShwaimi

**Affiliations:** Restorative Dental Sciences Department, College of Dentistry, University of Dammam, 7440 AlHosam, Dammam 32222-4371, Saudi Arabia

## Abstract

*Aim*. We surveyed the uptake of nickel-titanium rotary files (NTRFs) among all dentists in Saudi Arabia. *Methodology*. A questionnaire encompassing endodontic performance and NTRF uptake was e-mailed to all members of the Saudi Dental Society. Data were collected from participants during a three-month period and were analyzed using *χ* ^2^ tests and correlation coefficients. Level of significance was set at *P* = 0.05. *Results*. The overall response rate was 30.6% (*n* = 490), and 82.9% were found to perform root canal treatment (RCT). Among the 406 RCT performers, general dentists formed the bulk (45%). Among endodontists, 91.5% were using NTRF (*P* < 0.001). Those who graduated between 1991 and 2000 used NTRF more than any other group did (78.4%, *P* = 0.05). Graduates from Europe and Australia used NTRF most frequently (100%, *P* = 0.001), followed by those from North America (87%, *P* = 0.001), and finally by Saudi Arabian graduates (68.7%). Male respondents performed more endodontic procedures and used NTRF significantly more often than female respondents did (males: 73%; females: 56.2%) (*P* = 0.001). The most significant reasons for not using NTRF were “unavailability” (64.7%, *P* ≤ 0.05) and “lack of experience” (54.1%, *P* ≤ 0.001). *Conclusions*. We found that NTRF usage was not as widespread in Saudi Arabia as in other developing countries. Therefore, we suggest an improved implementation of NTRF in undergraduate and postgraduate curriculums and the provision of educational courses with a greater focus on this development.

## 1. Introduction

There have been many developments in endodontics since the year 1990, the most prominent of which has been the introduction of nickel-titanium rotary files (NTRFs), which were introduced by Walia et al. to overcome the rigidity of stainless-steel files, particularly those in large sizes [1, 2]. It is known that nickel-titanium instruments possess many other favourable characteristics compared with stainless-steel files, such as resistance to torsional fracture and lower modulus of elasticity [1, 3]. Several publications reported fewer procedural errors while preparing the root canal system using NTRF [4].

The adoption of any new technology is prone to user acceptance or rejection. Questionnaires are widely acknowledged as being a useful tool for identifying and collecting user feedback since they are objective, deliver results quickly, and are inexpensive. This tool helps in identifying the problems facing the target group and therefore helps to find solutions.

There have been many reports regarding NTRF and their properties, but few studies have investigated the adoption and usage parameters of these files. However, some surveys have been conducted in several countries, including Sudan [5], Denmark [6], Australia [7, 8], Belgium [9], Sweden [10], and, recently, in the United States of America [11].

Although NTRFs were introduced in Saudi Arabia in the early 1990s, thus far, there has been no information available regarding the extent of usage or uptake of these files by general dentists or endodontists. However, such information is considered critical for records, planning continuous education courses, and focusing on the weak links that dentists are facing. Therefore, the results can be used by the Saudi Endodontic Society and the Saudi Dental Society as reference and guidance as well as by the companies that produce NTRF to give them an improved understanding of the usage and adoption of the technique. The aim of this study was to survey the performance of root canal treatment (RCT) and usage of NTRF among a sufficiently representative number of dental students, dentists, endodontists, and other dental specialists residing in Saudi Arabia.

## 2. Materials and Methods

### 2.1. Design of the Questionnaire

The questionnaire survey comprised nine questions exploring endodontic performance, participant demographics, and attitude towards NTRF usage. Some of the questions used in a study by Bird et al. [11] were utilized in this survey and expanded to cover most of the aspects related to NTRF usage, which was based on information from the most recent publications on NTRF and root canal preparation. Questionnaires were formulated in two languages, English and Arabic. A pilot questionnaire was given to five dentists and endodontists to evaluate their understanding of the survey and the clarity of the questionnaire. From their feedback, the questionnaires were refined into their final format, and their responses have been included in the overall results. The questions were constructed in a manner that avoids leading the participants to a particular answer. For some questions, when a list of possible answers was given, participants were asked to choose the answer that best fitted their clinical situation, and if none of the selections were suitable, they were permitted to type an answer of their own.

One question had the option of free text only, but the remaining eight questions were constructed by using check boxes that were to be ticked appropriately next to each answer and an “others” option, which allowed for the typing of answer if selected. Selection of more than one answer was allowed for some questions, depending on the targeted idea. An explanation of the objectives of the study accompanied the questionnaires and assured confidentiality. In order to guarantee anonymity, the survey did not include the names or identification numbers of the participants.

### 2.2. Distribution and Collection of the Survey

Online questionnaires were distributed using the web interface “Survey Monkey.” Responses were collected over a three-month period; two reminder emails were sent to all responders, encouraging them to participate in the study. The survey was sent to a total number of 1620 members, following exclusion of inactive addresses on the Saudi Dental Society email database. Confidentiality was assured by asking all participants to ensure that they did not write their names, or anything else related to their identity, in the survey.

### 2.3. Data Analysis

If more than 5% of respondents had provided the same response to a question with an open text field at the time of analysis, then that answer was integrated into the data set for statistical analysis as a new category. Answers provided less frequently than 5% were stored as full text but were not analyzed in this study.

An SPSS package (SPSS Inc., Chicago, IL, USA) was used for statistical analysis. *χ* ^2^ tests and tests for differences in proportions were used to analyze the data set. The level of significance was set at *P* = 0.05; outcomes of statistical tests were only reported when this level was reached.

## 3. Results

The respondents were classified into the following categories.

### 3.1. Legible Participants

These formed the bulk of the participants and included all dental students, endodontic residents, endodontists, dentists, individuals with advanced education in general dentistry, and all the other dental specialties that were practicing in Saudi Arabia during the time of study. The total number of legible participants was 490.

### 3.2. Illegible Participants

These included any of the specialties mentioned in “legible participants” who were not practicing in Saudi Arabia while the study was being conducted. The total number of illegible participants was 23.

Any partially filled survey was disregarded and considered as “no response.” All specialties were categorized into four groups according to the skills and requirements necessary for that particular specialty.

The initial number of participants was 1620; however, after excluding those who were illegible, the final number was 1597, with a response rate of 30.6%.

### 3.3. Performance of RCT according to Specialty

Participants were asked if they currently perform RCT. This question was considered as key for the survey, since any participant who gave a negative response was directed to exit the survey at this point. As shown in Table 1, 406 (82.9%) of the 490 respondents were performing RCT. Almost 45% of the total numbers of dentists performing RCT were general dentists, but the highest percentage of those conducting within each group *per se* was observed in the Advanced Education in General Dentistry (AEGD) and Saudi Board in Restorative Dentistry (SBRD) group, followed by the Endodontists and Saudi Board in Endodontics groups (*χ* ^2^ = 95.36, *P* < 0.001), in this group only two participants answered by a negative response. The group that performed the least amount of RCT, excluding the dental students, was the “others” group, which comprised participants from all the other dental specialties that were not included in any of the other groups (Table 1).

### 3.4. Usage of NTRF in relation to Dental Specialties

Those participants who gave positive responses to the first question were asked to proceed to the second question: “Do you use NTRF?” As shown in Table 2, of the total number of dentists performing RCT (*n* = 406), 67.2% (*n* = 273) were using NTRF at the time of the survey, and of 273 participants who used NTRF, 103 (37.7%) were general dentists.

Interestingly, 97.1% of the participants in the AEGD and SBRD group were using NTRF, but this fell to only 91.5% in the Endodontists and Endodontic Residents group (*χ* ^2^ = 77.53, *P* < 0.001). Conversely, 75.5% of all participants representing other dental professions (prosthodontists, periodontists, pedodontists, orthodontists, oral surgeons, and others) used NTRF whenever they performed RCT.

### 3.5. Usage of NTRF in relation to Clinical Experience

In this question (Table 3), participants were asked about their clinical experience and were then classified into five uniform classes, related to time of graduation, with each class spanning 10 years. It was clearly shown that the majority (68.2%) of RCT was conducted by junior participants (2001–2010 group). Cross-tabulation was used between years of graduation and use of NTRF. There was a significant increase in NTRF usage with younger graduates, having less experience. The *χ* ^2^ test was used to check dependency between clinical experience and use of NTRF. A significant relationship (*χ* ^2^ = 7.80, *P* = 0.05) was found between these two variables for those in the 1991–2000 graduation group.

### 3.6. Usage of NTRF in relation to Place of Graduation

Of the 406 dentists who reported performing RCT, almost half of them had graduated in Saudi Arabia. The results showed that 145 (68.7%) of the Saudi graduates were using NTRF. All the participants (100%) who had graduated in Europe and Australia (*n* = 23) were NTRF users (*P* = 0.001). Of the 23 dentists who had graduated in North America 20 were NTRF users (*P* = 0.001), and of the 118 dentists who had graduated in Asia (primarily Syria, Lebanon), 66 (63.5%) were using NTRF. Conversely, those who had graduated in Africa used NTRF the least of all dentists surveyed. The differences between the proportions of those who did or did not use NTRF were statistically significant when considering place of graduation (*χ* ^2^ = 19.64, *P* = 0.001). 

### 3.7. Gender Effect

Table 5 shows that, of the 406 dentists performing RCT, 263 (65%) of them were male and 143 (35%) were female. There was a significant effect of gender on NTRF use; 73% of male dentists used these files versus only 56.2% of female dentists (*χ* ^2^ = 11.26, *P* = 0.001). 

### 3.8. Usage of NTRF in relation to Type of Practice

When respondents were asked about the type of practice in which they were working, it was found that 24% (*n* = 96) of those who were performing RCT were practicing at the Ministry of Health (Table 6). However, when we assessed each group independently, the highest usage of NTRF (*n* = 46, 93.9%) (*P* < 0.001) was observed at the Ministry of Defense Hospitals, followed by the Ministry of Interior Hospitals and the Ministry of National Guard Hospital (*n* = 11, 84.6%; *n* = 15, 88.2%; resp.). While an unexpected 55.8% (*n* = 48) of the participants practicing at the universities used NTRF, these files were used by 72% of the respondents from private hospitals and private dental clinics (*n* = 18 and *n* = 51; resp.). 

### 3.9. Usage of NTRF in relation to Their Location

When the participants were asked about their current workplace location, 397 out of 406 dentists gave information regarding the city in which they were working. Only nine participants chose not to disclose this information. We divided the country into five regions according to the population. As expected, 47.3% of all participants who performed RCT were located in the central region (with the largest population) (Table 7). NTRF use was highest in the northern region (77.8%) although this finding was not significant, second-highest in the central region (72.9%), and lowest in the eastern region (*n* = 43; 58.9%). 

### 3.10. Reasons Preventing the Usage of NTRF

When participants were asked about what may prevent them from using NTRF, they were permitted to choose more than one answer from a list of 11 possible reasons (Table 8). The group of participants who never used NTRF reported that the main reason for this was it “not being available” to them (64.7%, *P* ≤ 0.05). This was followed by “lack of experience” (54.1%, *P* ≤ 0.001) and “lack of knowledge or continuous education” (29.3%, *P* ≤ 0.05). Conversely, the group of participants who were using NTRF at the time of the survey reported that they are satisfied and that “there is no reason preventing them from usage” (41.8%, *P* ≤ 0.001). However, they also reported that if there was any reason that may prevent them from using NTRF in the future; it would be “fear of breakage” (30.8%, *P* ≤ 0.001), followed by “being expensive” (19.4%, *P* ≤ 0.001) (Table 8). 

## 4. Discussion 

There have been several publications on NTRF usage in several countries, including Australia [7, 8], Sudan [5], Denmark [6], Belgium [9], Sweden [10], and, more recently, in the United States of America [11]. However, unfortunately no such information exists with regard to Saudi Arabia, which is considered one of the biggest markets for NTRF in the Arabic Gulf region. Therefore, the aim of this study was to collect and analyze data regarding the demographics and usage of NTRF among dentists residing in Saudi Arabia. In contrast with the study by Parashos and Messer [7], in which the surveys were mailed to the target group, we utilized online questionnaires because we considered that mail may not be a reliable method of questionnaire distribution in Saudi Arabia. Moreover, use of an online survey is a fast and cost-effective way to distribute data to a relatively large target group and offers simple collection and analysis of feedback. Furthermore, the study investigator(s) can easily follow up with the target group and can send reminder emails to those who did not complete the survey. This kind of survey has a unique advantage over mailed hard-copy questionnaires; in that, in the online version, the participant cannot go to the next question without answering the present one. Moreover, all answers must be completed in order to submit the survey. However, one of the biggest disadvantages of online surveys is that they exclude participants who do not have emails, and/or those who do not open their emails at the time of the survey, which may result in biased outcome. 

In our study, we achieved a 30.6% response rate from the large number of dentists practicing across different regions of the country. This is close to the response rate achieved by Bird et al. [11]. Although the response rate was relatively low in our study, the total sample number of targeted candidates was larger than that used in most of the previous studies [6, 11]. Moreover, our response rate was higher than some studies with a large sample size, for example, that conducted by Slaus and Bottenberg (*n* = 4545) in which the response rate was 25% [9]. 

In order to achieve our aim of assessing the adoption and usage of NTRF among dentists residing in Saudi Arabia, we initially had to ask them if they perform endodontic procedures. 

We covered a wide array of specialties; therefore, in order to make the analysis simpler, and groups that have similar postgraduate level requirements were merged together, as seen in Table 1. 

The percentage of practitioners in our study who performed endodontic treatment was 82.8% (*n* = 406) of all the participants (*n* = 490) and almost 90% of general practitioners (Table 1). Our results were similar to a previous study that was conducted in Illinois, United States of America, where 90% of the dentists investigated performed RCT [12]. However, we reported higher RCT performance compared to that of other developing countries, for example, 67% in Kenya [13]. In a study conducted in Sudan, 85% of the dentists that were studied conducted endodontic treatment; however, the sample size was only 55 dentists, all of whom were working in one city [5]. In Table 1, two participants within the Endodontists and the Saudi Board in Endodontics group reported that they were not performing RCT. When we reviewed the demographics, we noticed that they were senior practitioners. Since our exclusive criteria did not include Endodontists who is not currently practicing, we included them in the statistics. We might think that they may have stopped practicing because of retirement or being occupied with other tasks such as administration. 

In our study, of the entire sample that performed endodontic treatment, 67.2% were NTRF users. However, when examining within the subgroups, we found that 52% of the general dentists and 91.5% of the Endodontist and the Saudi Board in Endodontics used NTRF. The Saudi Board in Endodontics is a four-year residency, in which the residents are asked to submit double the requirement for any two-year endodontic program. The percentage of NTRF usage across all dentists in Saudi Arabia is considered low when compared to NTRF usage among dentists and endodontists in the United States of America, which is 84% and 98%, respectively [11, 14]. However, our results compared favourably with those of some other countries, such as Finland, where only 28% of the 309 dentists use NTRF solely for shaping the canals of the teeth [14]. The AEGD and Saudi Board in Restorative Dentistry are two- and four-year residency programs, respectively. Both these programs have the same requirements as any endodontic program accredited by the American Association of Endodontists; therefore, the usage of NTRF was considerably high (97.1%) in this group. However, that might not explain why this group had higher usage of NTRF than the Endodontist group. Therefore, we felt it was necessary to identify the reasons that prevented the two endodontists (out of 32 endodontists) from using NTRF and found that the primary ones were unavailability of the NTRF and fear of breakage. Another possible reason is the effect of seniority and the difficulty of adopting a new technique. The three participants from the Saudi Board in Endodontics group who did not use NTRF reported that the main reasons for this were lack of experience or unavailability. We then assessed the adoption of NTRF by different participants in relation to their experience. We found that 68.2% of dentists performing RCT were considered young and fell into the “10 years' experience” or “recently graduated” groups (Table 3). Our results were in accordance with the demographics of the country, since 32% of the Saudi population is under the age of 15 and 62% of the population is between the age of 15 and 60. Furthermore, merely over 15 new dental schools have been established in the last 10 years. 

When we monitored the effect of years of experience on the usage of NTRF, we found that there was a significant reduction in the use of NTRF between 1991 and 2000 and 2001 and 2010—78.4% and 63.5%, respectively (*χ* ^2^ = 7.80, *P* = 0.05; Table 3). 

Parashos and Messer have shown a similar pattern of reduction of NTRF use from the decade 1981–1990 (31%) to the decade 1991–2000 (17%) [7]. In both this study and our own, the reduction in usage was significant among less-experienced dentists. 

Our results showed a significant relationship between place of graduation and usage of NTRF—*P* = 0.001 (Table 4). All 23 dentists who graduated in Europe or Australia used NTRF. We cannot explain the reason for this at the present time, but we believe that the manufacturing of NTRF in Europe may have had an impact on dental practice within that continent and therefore on these results. 

We then tested the relationship between gender and performance of endodontic treatment and found a significant difference; 86.8% of the male doctors performed RCT versus 75.4% of the females (Table 5) (*P* = 0.001). There was a significant difference between males and females for NTRF usage (73%, 56.6%; resp.) (*P* = 0.001). Currently, we cannot find an explanation for the effect of gender on RCT or NTRF. 

We did not expect to find that more than 45% of the participants working at universities were not using NTRF (Table 6). Therefore, we checked the curriculum of all the endodontic courses in those universities and found that NTRF was introduced only in the last 2-3 years. Conversely, over 90% of dentists working in military hospitals were using NTRF. A possible rationale for this is the existence of an Advanced Education for General Dentist's program, which is being supervised by endodontists, in military hospitals. Another possible explanation is the similarity between their requirement and the endodontic certificate accredited by the American Association of Endodontists. 

Universities and manufacturing companies should collaborate to have formal and informal communication with the end users in order to overcome the reasons for not using NTRF. This can be achieved through several continuing educational courses distributed among all the regions and focusing on regions in which use is particularly low. Furthermore, manufacturing companies should encourage new users by offering affordable, discounted prices. We did not cover the effect of education courses on the adoption of NTRF in terms of quantity and quality, so further studies are needed to evaluate such an effect. Our results indicate the need for more hands-on workshops and lectures to increase the confidence of dentists with regard to overcoming the fear of breakage. It appears that both the Saudi Dental Society and the Saudi Endodontic Society should address these issues in the dental societies and dental schools to improve skills and user acceptance. 

## 5. Conclusion 

This paper surveyed the endodontic performance and attitude towards NTRF uptake by different groups of practitioners residing in Saudi Arabia. Within the limitations of this study, we found that NTRF usage was not as widespread as in other developing countries. We suggested that collaboration between universities, dental societies, and manufacturing companies would lead to improved implementation of NTRF in undergraduate and postgraduate curriculums, as would the provision of more focused educational courses. Further studies are needed to evaluate the effect of these courses on the usage of NTRF. There was a clear effect of gender on endodontic treatment performance and NTRF usage, which may indicate a serious implication for the future, one that warrants further investigation to ascertain the reasons behind it. 

## Figures and Tables

**Table 1:** Root canal performance according to specialty.

Specialty	Participants who perform RCT	Perform RCT	No. RCT (%)^a^
Dental student	43	31 (72.1)	12 (27.9)
General dentist	220	198 (90)**	22 (10)
AEGD^b^ & Saudi board in restorative dentistry	71	69 (97.2)**	2 (2.8)
Endodontist & Saudi board in endodontics	61	59 (96.7)**	2 (3.3)
Other specialties	95	49 (51.6)	46 (48.4)

Total	490	406 (82.9)	84 (17.1)

^a^The percentage valued between parenthesis refers to each group, **(*χ* ^2^ = 95.36, *P* < 0.001), ^b^Advanced Education in General Dentistry (AEGD).

**Table 2:** Response details for NTRF usage according to specialty.

Specialty	Use NiTi (%)^a^	Do not use NiTi (%)^a^
Dental student	12 (38.7)	19 (61.3)
General dentist	103 (52)	95 (48)
AEGD^b^ & Saudi board in restorative dentistry	67 (97.1)**	2 (2.9)
Endodontist & Saudi board in endodontics	54 (91.5)**	5 (8.5)
Other specialties	37 (75.5)	12 (24.5)

Total	273 (67.2)	133 (32.8)

^a^The percentage valued between parenthesis refers to each group, **(*χ* ^2^ = 77.53, *P* < 0.001), ^b^Advanced Education in General Dentistry (AEGD).

**Table 3:** Response details for NTRF and year of graduation.

Years	Participants who perform RCT	Use NiTi (%)^a^	Do not use NiTi (%)^a^
1971–1980	6	3 (50)	3 (50)
1981–1990	35	25 (71.4)	10 (28.6)
1991–2000	88	69 (78.4)*	19 (21.6)
2001–2010	277	176 (63.5)	101 (36.5)

^a^The percentage valued between parenthesis refers to each group, *(*χ* ^2^ = 7.80, *P* = 0.05).

**Table 4:** Response details for NTRF usage and place of graduation.

Place of graduation	Participants who perform RCT	Use NiTi (%)^a^	Do not use NiTi (%)^a^
Saudi Arabia	211	145 (68.7)	66 (31.3)
North America (USA, Canada)	23	20 (87)**	3 (13)
Europe and Australia	23	23 (100)**	0 (0)
Africa	31	16 (51.6)	15 (48.4)
Asia	104	66 (63.5)	38 (36.5)
Others	14	3 (21.4)	11 (78.5)

^a^The percentage valued between parenthesis refers to each group, **(*χ* ^2^ = 19.64, *P* = 0.001).

**Table 5:** Response details for RCT performance and NTRF in relation to gender.

Gender	Participants who perform RCT	Use NiTi (%)^a^	Do not use NiTi (%)^a^
Male	263	192 (73)**	71 (27)
Female	143	81 (56.6)	62 (43.4)

^a^The percentage valued between parenthesis refers to each group, **(*χ* ^2^ = 11.26, *P* = 0.001).

**Table 6:** Response details for NTRF usage and type of practice.

Place of practice	Participants who perform RCT	Use NiTi (%)^a^	Do not use NiTi (%)^a^
University	86	48 (55.8)	38 (44.2)
Ministry of Health Hospital	96	58 (60.4)	38 (39.6)
Ministry of Defense Hospital	49	46 (93.9)**	3 (6.1)
Ministry of Interior Hospital	13	11 (84.6)	2 (15.4)
National Guard Hospital	17	15 (88.2)	2 (11.8)
Private Hospital	25	18 (72)	7 (28)
Private Dental Clinic	71	51 (71.8)	20 (28.2)
Medical Polyclinic (has dental clinic)	39	21 (53.8)	18 (46.2)
Others	10	5 (50)	5 (50)

^a^The percentage valued between parenthesis refers to each group, **(*χ* ^2^ = 33.56, *P* < 0.001).

**Table 7:** Response details for NTRF usage according to region.

Region of practice	Participants who perform RCT	Use NiTi (%)^a^	Do not use NiTi (%)^a^
Central	188	137 (72.9)	51 (27.1)
West	107	69 (64.5)	38 (35.5)
East	73	43 (58.9)	30 (41.1)
South	20	13 (65)	7 (35)
North	9	7 (77.8)	2 (22.2)

^a^The percentage valued between parenthesis refers to each group, no significant finding (*χ* ^2^ = 5.88, *P* = 0.2).

**Table 8:** Reasons preventing the usage of NTRF.

Reasons preventing the usage of NiTi	Users of NiTi (%)^a^	Never used NiTi^b^ (%)^a^
Not available	52 (19)	86 (64.7)*
Lack of experience	23 (8.4)	72 (54.1)**
Lack of knowledge or continuous education	18 (6.6)	39 (29.3)*
Fear of perforation	24 (8.8)	17 (12.8)
Fear of breakage	84 (30.8)**	15 (11.3)
Too expensive	53 (19.4)**	11 (8.3)
Do not see an advantage over hand files	10 (3.7)	8 (6)
Difficult to learn and use	1 (0.4)	6 (4.5)
Not seeing a lot of patients needing root canal treatment	2 (0.7)	5 (3.8)
No reason preventing me to use them	114 (41.8)**	5 (3.8)
Other reasons	7 (2.6)	5 (3.8)

^a^The percentage valued between parenthesis refers to each group, Significant at **(*P* ≤ 0.001); *(*P* ≤ 0.05), ^b^Reasons preventing NTRF usage among nonuser group in descending order.
